# The perceptions of nurses and physicians in primary care of rehabilitation for frail older adults

**DOI:** 10.1177/02692155241258286

**Published:** 2024-06-02

**Authors:** Maria Laitalainen Törnudd, Anneli Peolsson, Maria M Johansson

**Affiliations:** 1 Department of Health, Medicine and Caring Sciences, Unit of Physiotherapy, 4566 Linköping University, Linköping, Sweden; 2 Department of Rehabilitation in Norrköping, and Department of Health, Medicine and Caring Sciences, 4566 Linköping University, Linköping, Sweden; 3 Department of Occupational and Environmental Medicine Centre, Department of Health, Medicine and Caring Sciences, Unit of Clinical medicine, 4566 Linköping University, Linköping, Sweden; 4 Department of Activity and Health, and Department of Health, Medicine and Caring Sciences, 4566 Linköping University, Linköping, Sweden; 5 Department of Acute Internal Medicine and Geriatrics in Linköping, and Department of Health, Medicine and Caring Sciences, 4566 Linköping University, Linköping, Sweden

**Keywords:** Frail elderly, occupational therapy, patient care team, physical therapy specialty, primary care

## Abstract

**Objectives:**

To investigate the perceptions of primary care nurses and physicians of the potential contributions of physiotherapists (PTs) and occupational therapists (OTs) in the treatment of frail older persons, as well as the obstacles to, and opportunities for, collaboration.

**Design:**

A qualitative study.

**Participants and setting:**

Nurses (n = 9) and physicians (n = 8) in primary care in the county council [14 women (82%)] with experience working with older people.

**Method:**

Interview study conducted with a semi-structured interview guide. Analyses were carried out with content analysis with an inductive approach.

**Results:**

The analysis resulted in six categories: knowledge of physiotherapy and occupational therapy interventions; what triggers the need for physiotherapy and occupational therapy?; the availability of rehabilitation interventions; teamwork opportunities and difficulties; motivating the patient; the site of the rehabilitation.

**Conclusions:**

Close and clear collaboration between nurses and physicians and PTs and OTs is an important factor in ensuring that rehabilitation interventions provide the greatest possible benefit to the patient. Improving communication between different healthcare providers and clarifying the contact routes is a prerequisite for patients to be able to get the rehabilitation they need. More research is needed to determine the best approach to achieving this goal.

## Introduction

Demographic changes will present a major challenge for the support, care, and nursing of older people. It is of great importance to make the best use of all healthcare providers. Physical ability, such as muscle strength, balance, and general fitness, decreases with age. Diseases of old age can result in pain and severely impaired physical function. This increases the risk of falling, illness, and suffering, and thereby increased morbidity and mortality.^1^ Ek et al.^2^ showed that it is possible to predict an elevated risk of falling up to 10 years before the fall, which suggests that there are great opportunities for primary prevention. The risk of frailty increases with age, and this should be considered both for preventative and rehabilitative interventions. Frailty has been defined by the World Health Organisation (WHO) as ‘a clinically recognisable state in older people who have increased vulnerability, resulting from age-associated declines in physiological reserve and function across multiple organ systems, such that the ability to cope with every day or acute stressors is compromised’.^3^ It is important that individuals within the group of frail older people are assessed and then receive individually selected interventions by physiotherapists (PTs) and occupational therapists (OTs)^4–10^ if necessary.

Primary care nurses and physicians frequently encounter older adults and need to have knowledge of when to refer patients to other professions. In the county council of Region Östergötland in the southeast part of Sweden (ca 470,000 inhabitants), where this study took place, special elder care clinics (*äldrevårdsmottagning*) for older people (75+) were introduced at some of the healthcare centres in 2019 and 2020. The teams at the elder care clinics mainly consist of nurses and a lead physician. Other professions, such as PTs and OTs, are referred to when needed. The elder care clinics are based on the results of a previous study on ‘proactive healthcare for frail elderly persons’.^11^ The working model was intended to promote a more proactive approach in primary care for frail older persons and resulted in fewer days in hospital for the intervention group.^13^ However, the results of this intervention also indicated that few individuals were referred to PTs and OTs.^14–16^ Nurses and physicians in primary care generally have some knowledge of when a referral to an OT or PT is appropriate and what rehabilitation services are available, but this level of knowledge may vary. Previous studies have explored nurses’ and physicians’ perceptions of physiotherapy, occupational therapy, and the role of the PT and OT in different areas.^17–25^ But to our knowledge, no studies to date have investigated the knowledge of nurses and physicians regarding the benefit of physiotherapy and occupational therapy in primary care interventions aimed at frail older individuals. The aim of this study was therefore to investigate nurses’ and physicians’ perceptions of what physiotherapy and occupational therapy can contribute to primary care for frail older adults and the obstacles to, and opportunities for, collaboration.

## Methods

The design was a qualitative interview study with inductive content analysis.^26^ The interviews were conducted individually with semi-structured and open-ended questions.

### Participants and setting

The informants worked at healthcare centres in a county council (Region Östergötland) in the southeast part of Sweden. At the time of the study, some of the healthcare centres had started a special elder care clinic for older people, with nurses who focus on the needs of older patients. Most of the healthcare centres do not have rehabilitation professionals working at the healthcare centre. The inclusion criteria were working as a nurse or physician in primary care and having experience working with older patients. The managers of 18 public healthcare centres (of 33 in total) within the county council and two (of 10 in total) private healthcare centres in the same area were contacted by email for approval to conduct interviews with the employees in question. The healthcare centres were chosen to ensure a mix of urban and rural settings and a diverse representation of centres, as some centres have implemented a special elder care clinic, and some have not. Managers from 13 of the public healthcare centres in the study area and one private healthcare centre replied and sent the invitation for participation to staff members working with older people. At these healthcare centres, it is common for 1–3 staff members to work more directly with the group of frail older individuals. All the nurses and physicians (informants) who responded to the invitation and wished to participate in an interview and met the inclusion criteria were included. Participants were enrolled after informed consent was obtained. They first received written information about the study by email. Oral information was also given before the interview started. No further definition of frail older individuals was communicated. The interviews were conducted digitally by the first author between May 2021 and March 2022. The authors designed an interview guide with questions regarding the rehabilitation of frail older people (which is available on request). The guide included questions such as, ‘What do you think PTs and OTs can contribute for these patients?’, ‘When is it relevant to refer patients to a PT/OT?’, ‘What do you think would be needed for an optimal collaboration with a PT/OT?’ All interviews ended with an open question asking, ‘Is there anything more you wish to share on this subject?’.

A pilot interview was conducted with a nurse at the nearest healthcare centre to determine how well the questions corresponded to the purpose of the study before starting data collection. In order to obtain answers with more depth and richness, the interview guide was clarified by the author and one of the co-researchers after the pilot interview by asking the same open-ended questions in a few different ways. The pilot interview was included in the analysis. All interviews were recorded (sound) and transcribed verbatim. Seven of the interviews were transcribed by the author and the rest by a professional transcription company. Only the first author of the study (interviewer) knew the names of the informants. The transcribed interviews and the recordings are stored at the University of Linköping, and only involved researchers have access to these. The data has been pseudonymized, and each informant has been assigned a code. The data has been presented at a group level in a way that the identities of the informants could not be disclosed. The data has been handled according to the General Data Protection Regulation.

### Data analyses

The collected data were analysed through a qualitative content analysis according to Graneheim and Lundman.^27^ The analysis was carried out using an inductive approach, which means that the text was analysed as openly as possible to find similarities and differences in the data. The text was read several times by the authors, and open encoding was performed by MLT and MJ. The codes were grouped and categorised in dialogue between the three researchers. One of the researchers works in geriatrics (OT) and has an educational background in qualitative research (PhD); one researcher works in primary care (PT and MSc); and one researcher, a university professor, has previous clinical experience in primary care (PT). Please see Table 1 for the coding procedure.

**Table 1.** Coding procedure. Examples of meaning units, condensed units, codes, and categories.

Meaning unit	Condensed unit	Code	Category
… how do you get hold of it, where do I call, should I call myself, or is it someone else? I mean, if I as a caregiver, can I send a referral or should I call… or is the patient expected to call? Who will make the contact? (Informant 6)	Where do I call? Can I send a referral, or should I call, is the patient expected to call?	Difficulties contacting rehab	The availability of rehabilitation interventions
On the one hand, I would think it was perhaps good to have some kind of meeting on site, or the link; that is, with, that you get information about what can be offered. And which patients you would like to see, or help. (Informant 8)	Works well to meet on site or via link, get information about what can be offered. Which patients you want to see, help.	On-site meeting or link Cooperation Information	Teamwork—opportunities and difficulties

## Results

A total of 17 persons (9 nurses and 8 physicians) were interviewed. The informants were aged between 33 and 63 years (mean 47.7) and consisted of 14 women and 3 men. They had worked between 7 and 44 years (median 17) in their professions, and this was also the number of years they had worked with older individuals. The interviews lasted between 18 and 41 min (mean 27). The analysis resulted in six categories: knowledge of physiotherapy and occupational therapy interventions; what triggers the need for physiotherapy and occupational therapy; the availability of rehabilitation interventions; teamwork opportunities and difficulties; motivating the patient; the site of rehabilitation. The results are presented under each category. Coded quotations are presented to illustrate each category.

### Knowledge of physiotherapy and occupational therapy interventions

In general, all informants had relatively good knowledge of what PTs and OTs can contribute to the care of frail older persons. Two of the informants felt that physiotherapy and occupational therapy were closely related professions in terms of responsibilities and the interventions offered. Several informants believed that PTs and OTs could offer alternatives to surgery and that there were other ways to reduce pain and make life easier. One of the informants perceived that PTs offered better examination techniques than his/her own professional group. Some reported that PTs and OTs were good at detecting healthy aspects in the patient and that the patient gained greater independence through rehabilitation, enabling the patient to live at home longer. The fact that PTs and OTs could, if necessary, educate caregivers, home health workers, and relatives was seen as positive. Assistive devices for various difficulties, diseases, and fall prevention were consistently understood to be available interventions. Some difficulties that were more frequently highlighted by informants, and where assistive devices were noted as being able to make a difference, were sitting difficulties, eating difficulties, and cognitive impairments. A strong sentiment in the data was that healthcare providers working with older people should maintain a broad understanding of their own options and responsibilities when delivering care, and that it is possible to offer different actions, depending on the patient's needs. Moreover, the informants provided the following examples of available interventions: housing modifications; ADL assessment; assessment of grip ability and fine motor skills; assessment of mobility; identification of problems; osteoarthritis school; compression stockings; pain relief; training, such as strength and gait training; encouragement for training/activity; advice on movement techniques; acupuncture; testing of orthotics; dementia assessment; ergonomics; and counselling, for example, on posture.

I think they can contribute a lot. It has a lot to do with assistive devices, but also really with training, and maybe I have missed that we don't collaborate even more in that area. Just trying to maintain function and things like that. (Informant P5)

Despite a generally good understanding of the interventions PTs and OTs provides for frail older people, the knowledge of nurses and physicians was also lacking in certain areas. There was uncertainty about the difference between rehabilitation at home and at the clinic, and on whose initiative, rehabilitation was initiated. Most informants wanted to know more about what physiotherapy and occupational therapy can offer. Some informants wanted to gain more knowledge about the training, goals, and purpose of OTs and PTs.

### What triggers the need for physiotherapy and occupational therapy?

The informants expressed that the need for rehabilitation interventions became more evident when the patient expressed difficulties with balance or had known walking difficulties, difficulty grasping things with his/her hands, dizziness or pain, or had simply lost different abilities. The need for referral also emerged when it came to maintaining or improving function due to muscular problems; stiffness; problems in the neck, back, hip, knee, elbow, and hand; problems in the lower abdomen; and difficulty sitting postoperatively. Referrals were also seen as appropriate when treating orthopaedic patients, in cases where cognitive investigation was needed, and when coaching patients to engage in physical activity or exercise. The need for rehabilitation could be observed during visits to the healthcare centre or during home visits. Some informants also described that it was important in their profession as a nurse or physician to be aware of the patient's needs and their need for rehabilitation.

Yes, the first thing that comes to mind are patients who have been discharged from the hospital that have sustained fractures or bone injuries. Also, patients who are seeking medical care for dizziness, patients we ask physiotherapists to help us with. Of course, also the patient who has had long-term pain – we sometimes ask physiotherapists to help us with them as well. (Informant P11)

Informants that have participated in the project ‘Proactive healthcare for frail elderly persons’ (n = 5),^11,28^ all used the Primary care Assessment Tool for Elderly (PASTEL)^12^ and felt that the tool had been helpful. The form facilitated the assessment of what interventions was needed for specific individuals, and it was easier to note balance problems, musculoskeletal disorders, ADL needs, mobility problems, the need for assistive devices, housing needs, exercise habits, and pain. It also made it easier to pinpoint the issue that was the most concerning for the individual.

When you do a medical round, you have different options to go through; what actions to do. We do a frailty assessment according to the Clinical Frailty Scale, and then we note, ‘OK, how fragile is the patient?’ Then we have certain interventions, among other things, functional assessment by the physiotherapist and activity assessment by the occupational therapist. (Informant P4)

Two of the informants stated that Senior Alert,^29^ a national quality register with risk assessment screening and follow-ups, could be a good complement to PASTEL,^12^ partly to obtain an even more comprehensive assessment of the frail older person and partly to better determine when a referral to a PT or OT is appropriate.

### The availability of rehabilitation interventions

The availability of rehabilitation interventions was a subject that emerged in several of the interviews. Some perceived that there was good accessibility in the smaller municipalities, while others reported poor accessibility due to frequent rejections of referrals. Most of the informants were unsure where to turn if rehabilitation interventions were needed. There was uncertainty about whether they should refer the patient to the PT or OT working in primary care in the county council, or if they should refer the patient to the municipality's rehabilitation team. There was also uncertainty about the procedure, that is, whether contact should be made by referral, phone call, or if the patient should contact the rehabilitation unit himself/herself. Most stated that it was common for them to be in contact with the rehabilitation team within the municipality.

And then you don't know if it's the county council you're going to turn to or if it's the municipality. (Informant P1)

Most informants made very frequent or frequent referrals to physiotherapy and occupational therapy, while some made referrals less frequently. Some felt that physiotherapy and occupational therapy were underutilised and that it was easy to forget to make a referral for rehabilitation. The reasons informants cited for lower referrals to physiotherapy were the perception that there were not enough resources for rehabilitation, that PTs and OTs do not have sufficient knowledge of the patient, or that there is poor continuity. Dissatisfaction with the frequent rejection of referrals to the municipality's rehabilitation team and a lack of time within the healthcare centre for older adults were cited as other factors contributing to reduced referrals.

Several informants expressed a desire for better accessibility and shorter wait times for rehabilitation appointments. Factors that were considered to affect accessibility included access to resources, prioritisation of time, organisational affiliation that increased the distance to care, and interest in working with frail older people. Informants stated that it was easier to communicate with other healthcare providers in the county council by using the messaging function in the digital medical record system. This was reported to be a convenient way to communicate with the municipality. Communication by phone was generally perceived to be more difficult because it requires synchronous communication.

It usually doesn't always work across the clinic boundary, but it works great. Then she writes what they have done, what the plan is, and I can give feedback when I will meet the patient and how it is going with the sick leave. It is a contact area that could also work when it comes to frail elderly individuals, as long as everyone is using Cosmic.* (Informant P6)

*the medical record system used by the county council

### Teamwork opportunities and difficulties

Teamwork was considered important in all interviews. Several informants described teamwork as a perishable commodity and stated that a long-term system is needed to facilitate continuous collaboration, which also requires personal knowledge of the other team members. An obstacle to teamwork that was mentioned in several of the interviews was difficulties associated with belonging to different organisations, which made it difficult to engage in dialogues with other providers, and different assessments were sometimes done for the same problem in the same patient. Another difficulty that was mentioned concerning working in different organisations was the use of different medical record systems.

…but they are a municipal unit, and we are county council unit, and it is also difficult. So, they do a lot, but we don't have the same journal system. We don't have, I can't go in and look, but you must call so it's more complicated to have a collaboration there. (Informant P4)

The cutoff for when a patient qualifies for home care was sometimes considered too strict and was therefore seen as a potential obstacle. Some informants reported that a lack of time was a major obstacle and that they rarely had time for feedback or discussion of patient cases. Many lacked the time for close cooperation with PTs and OTs. Several stated that there should be access to PTs and OTs at the healthcare centre for better contact and continuity, and to better enable them to discuss cases, particularly complex cases, and to have the opportunity to meet the patient together with other healthcare professionals. This could enable joint assessment and the development of a collaborative care plan. Informants stated that it was easier to make contact with an OT than a PT.

I would like us to have the opportunity to have more contact with the clinic here then, that is, occupational therapists and physiotherapists here at the healthcare centre. (Informant P2)

As part of the team at the healthcare clinic for older people, PTs and OTs were seen as a positive addition. Informants consistently reported a wish for team meetings. Two of the informants already participated in regular team meetings. The team meetings were perceived as important for bridging the learning divide between professions, for exchanging recommendations, and conveying information about what can be offered in the rehabilitation activities. It was considered important to have a clear agenda at team meetings. In general, team meetings worked better within the municipality, especially in the smaller municipalities. Meetings that included coordinated individual planning were perceived to work well, but still required a great deal of effort from participants. Suggestions for team meetings emerged, such as joint rounds, or meetings held in-person or digitally. Teamwork was seen as a means to increase the focus on the patient, with better follow-up and thus increased benefits for the patient.

I would think it might be good to have some kind of meeting on site or, like this, on the link, that is, with, that you get information about what can be offered. (Informant P8)

Other wishes were to have time allocated for patient appointments to be booked directly, to make greater use of the messaging function in the Cosmic medical record system, and to have established routines pertaining to collaboration. The medical record entries made by PTs were seen as a positive, as they were perceived to be well written and provided good information about the patient's symptoms, interventions, and results.

### Motivating the patient

The importance of the patient's motivation to participate in rehabilitation was an area pointed out by about half the informants. Patients were often perceived to be difficult to motivate, and it was reported that the nurse sometimes needed to convince patients to participate in rehabilitation by demonstrating the benefits. Several informants reported that patients could sometimes be hesitant to participate in rehabilitation. One explanation for this could be that patients have more faith in medications, although it was suggested that this may be a generational issue. Some informants described that patients found it difficult to have someone in their home, perhaps more so during the COVID-19 pandemic. Informants perceived that patients had a certain resistance to exercise, especially at the healthcare centre. Adherence to the rehabilitation plan was often considered to be better in at-home rehabilitation.

Some informants also felt that many patients do not understand the competence offered by PTs and OTs, and that they do not believe that exercise can make a difference, which one informant felt could be due to generational differences.

Right, yes, but I think you could make a pretty big difference really, but that, yes, but maybe it's hard because they don't know what they're going to expect. (Informant P3)

### The site of rehabilitation

The fact that PTs and OTs were able to make home visits was seen as very positive by all informants. Most patients had contact more often with the home rehabilitation team within the municipality. There were several benefits offered by home visits. The fact that clinicians could assess the patient in their home and more easily determine what the patient needs in terms of interventions made rehabilitation more individualised. Another advantage of home visits was that several professional categories could overlap. One of the informants expressed a wish that the hand therapy team could also make home visits. Some of the informants felt that the threshold for patients to receive help from a PT or OT was lower if there was an option for home visits. Rehabilitation training at home was more adapted to the home and to the patient's daily routines in the home. Several informants also noted some disadvantages to home rehabilitation, namely, that it was more time and resource intensive for PTs and OTs and created uncertainty for patients if they performed exercises alone, as no one checks whether the exercises are being performed correctly. It was also suggested that reliance on home health creates a greater risk of patient isolation.

Yes, it's the occupational therapist with assistive devices, and so on. And they make home visits to the home. I mean, that's the thing. I never work. I never see the patients at home. Without me seeing them, if I see them, it's at the healthcare centre, and otherwise it's through telephone contact, so to speak. But that's the advantage – that they make home visits and can make an assessment at home. So, that I think is fantastic. And the same with physiotherapists who can make home visits. (Informant P12)

The potential advantages of clinic visits include maintaining the patient's integrity, proximity to other professions (thus facilitating the option for combined visits), access to exercise equipment and assistive devices, smooth and resource-efficient use of PTs and OTs, time savings, and the ability to exercise in groups (making it a social activity for patients, which increases motivation). The disadvantages were difficulty getting to the clinic, that rehabilitation plans could be complicated by travel, and potential additional costs for the patient. A concern emerged that cognitively impaired patients often get caught between the county council and the municipality.

I would imagine that since we are such a large healthcare centre, we could offer two days a week, that you can come for different exercises and balance training, that you have the training in the house. So, it would be optimal for me at such a large healthcare centre, also for the patient, to come out and meet some other people and get to do this in a group. It does so much good. (Informant P4)

## Discussion

The results showed that the understanding of what PTs and OTs have to offer patients is not a significant problem, but there was a desire among nurses and physicians to engage in a closer and clearer collaboration with PTs and OTs to be able to use rehabilitation interventions in the best possible way. The availability of rehabilitation interventions was an area that appeared in several of the interviews. One of the reasons reported for uncertainty regarding where to direct patients for rehabilitation was that PTs and OTs often worked at a different facility, not at the healthcare centre. Another cause of uncertainty was that healthcare professionals belonged to different organisations (i.e. the county council and municipality), which was further complicated by different record systems. A study by Nelson et al.^20^ found that one of the barriers to referral was a lack of contact and communication with the PT, which speaks to the importance of clear contact routes and routines. A study by Knecht-Sabres et al.^24^ resulted in five themes concerning the underutilisation of occupational therapy. One theme was not having an OT present in the same setting. Several informants stated that contacting an OT was easier than contacting a PT. One reason for this may be that the OT within the municipality more often communicates with a nurse at the healthcare centre and is more likely to be involved in matters related to assistive devices and adaptations in the home. Improving communication and clarifying contact routes is a prerequisite for patients to be able to get the rehabilitation they need.

All informants highlighted teamwork as an important factor in creating better communication, which was also seen as a desirable outcome by all informants. Most informants lacked access to effective collaboration and a team approach that included PTs and OTs. Informants suggested that team meetings would be an effective way to discuss patient cases with each other, learn from each other, and get more information about what interventions are available. It was suggested that team meetings could be held either on site or via a weblink. Several studies have shown that regular teamwork is essential in the treatment of frail older adults. These studies have shown that teamwork centred around patients with complex needs, where many healthcare professionals are involved, is usually more effective than working separately.^30^ Regular meetings and good communication often lead to a better understanding between different professions and thus better conditions for good cooperation.^31^ A study by Nelson et al.^20^ on the role of physiotherapy in palliative care found that a majority of district nurses were reluctant to refer patients to physiotherapy, as they did not believe that PTs had adequate knowledge of palliative care and worried that PTs might provide patients with false hope. The study also noted a lack of contact and communication between the nurses and physiotherapy providers, as well as a lack of knowledge and experience in physiotherapy.^20^ This shows how important it is to have good communication between different professions and reinforces the importance of teamwork. Several studies have investigated teamwork between nurses and physicians, but fewer studies have included other professions. Regarding teamwork in primary care, it is important for professionals to work together and learn from each other in practice.^32^

Previous studies^21,22^ have shown that the general level of knowledge regarding occupational therapy is limited. A study from the United States^22^ showed a positive relationship between knowledge of occupational therapy and the number of referrals. Previous studies on the role of PTs^17,18^ showed that knowledge of physiotherapy was relatively good. To the authors’ knowledge, the study by Åberg et al.^25^ is the only Swedish study that overlaps with the area investigated in the present study. It examines how nurses and housing managers perceive their own professional function, the role of PTs and OTs in the care of older people, and teamwork and rehabilitation. PTs and OTs are described as experts, and that they bring competence that other healthcare professionals lack, as other studies have also shown.^17,18^ The presence of PTs and OTs facilitated communication and knowledge about rehabilitation and was an important factor in improving cooperation in rehabilitation in nursing homes. These findings align with what has emerged in the present study in terms of teamwork and accessibility.

A qualitative approach was chosen according to the aim of this study. Interviews allowed the authors to highlight the perceptions of nurses and physicians.^33^ The sample was well distributed, as there was variation in age, occupational category, number of working years, and geographical area. However, most participants were women, which can possibly be seen as a weakness. At the same time, most healthcare professionals in Sweden today are women. Seventeen interviews were considered sufficient, as there was saturation in content.^34^ For the best possible interview situation, it would have been desirable to hold the interviews in a direct meeting with the informants. As interviews were conducted during the Coronavirus pandemic, it was decided that the best option was to hold digital interviews instead. The interview guide was designed in the research group, which can be seen as a strength, as different aspects pertaining to questions and how they could best be asked emerged.^35^ The interviews were conducted by the first author, which can be seen as both a strength and a weakness. The content of the interviews could be influenced by the person who conducts the interviews, but at the same time, this provides better uniformity.^36^ Qualitative content analysis was chosen, as it is useful when reviewing different types of texts such as interviews. Furthermore, this approach allows interpretation to take place on different levels. The analysis was carried out with an inductive approach. The manifest content was analysed with a low degree of interpretation.^36^ The interview texts were read by three different researchers, and all were involved in the analysis process (triangulation). This can be seen as a strength, as it increases the chances that more thoughtful codes will emerge.^27^ The researchers’ pre-understandings were expected to effect the analysis, which can be both advantageous and disadvantageous. The disadvantage may be that the experiences of the researcher colour the analysis. But at the same time, this can be seen as a positive, as the researchers were more familiar with work with frail older people and thus have a better understanding of the subject matter. Pre-understandings can facilitate the generation of new knowledge and a deeper understanding of the subject matter. One of the researchers had limited experience working with older people, which, given the other researchers’ experiences, can be seen as a strength, as this researcher could approach the subject matter from another direction.^37^

In terms of transferability, the study is mainly valid for primary care in Sweden and in countries with similarly structured healthcare systems. Future studies should investigate which form of collaboration works best between physiotherapy, occupational therapy, and the elder care clinic, and in what ways PTs and OTs can best contribute when it comes to frail older people. One may conclude that close and clear collaboration between nurses and physicians and PTs and OTs is an important factor in being able to use rehabilitation interventions in the best possible way. Improving communication between different healthcare providers and clarifying the contact routes between them is a prerequisite for patients to be able to receive the rehabilitation they need.

**Clinical messages**

1. It is important to provide information to staff at healthcare centres about who to turn to for rehabilitation referrals for the frail elderly, and how.
2. Having professionals situated in different settings and organisations can hinder rehabilitation in frail older individuals.
3. There is a need to evaluate further holding team meetings with rehabilitation staff in primary care.
